# Genome-wide association study of alcohol consumption and genetic overlap with other health-related traits in UK Biobank (*N*=112 117)

**DOI:** 10.1038/mp.2017.153

**Published:** 2017-07-25

**Authors:** T-K Clarke, M J Adams, G Davies, D M Howard, L S Hall, S Padmanabhan, A D Murray, B H Smith, A Campbell, C Hayward, D J Porteous, I J Deary, A M McIntosh

**Affiliations:** 1 Division of Psychiatry, University of Edinburgh, Royal Edinburgh Hospital, Edinburgh, UK; 2 Centre for Cognitive Ageing and Cognitive Epidemiology, University of Edinburgh, Edinburgh, UK; 3 Institute of Genetic Medicine, Newcastle University, Newcastle upon Tyne, UK; 4 Institute of Cardiovascular and Medical Sciences, University of Glasgow, Glasgow, UK; 5 Aberdeen Biomedical Imaging Centre, University of Aberdeen, Aberdeen, UK; 6 Division of Population Health Sciences, University of Dundee, Dundee, UK; 7 Generation Scotland, Medical Genetics Section, Centre for Genomic and Experimental Medicine, Institute of Genetics and Molecular Medicine, University of Edinburgh, Edinburgh, UK; 8 MRC Human Genetics Unit, MRC IGMM, University of Edinburgh, Edinburgh, UK; 9 Department of Psychology, University of Edinburgh, Edinburgh, UK

## Abstract

Alcohol consumption has been linked to over 200 diseases and is responsible for over 5% of the global disease burden. Well-known genetic variants in alcohol metabolizing genes, for example, *ALDH2* and *ADH1B*, are strongly associated with alcohol consumption but have limited impact in European populations where they are found at low frequency. We performed a genome-wide association study (GWAS) of self-reported alcohol consumption in 112 117 individuals in the UK Biobank (UKB) sample of white British individuals. We report significant genome-wide associations at 14 loci. These include single-nucleotide polymorphisms (SNPs) in alcohol metabolizing genes (*ADH1B/ADH1C/ADH5*) and two loci in *KLB*, a gene recently associated with alcohol consumption. We also identify SNPs at novel loci including *GCKR*, *CADM2* and *FAM69C*. Gene-based analyses found significant associations with genes implicated in the neurobiology of substance use (*DRD2*, *PDE4B*). GCTA analyses found a significant SNP-based heritability of self-reported alcohol consumption of 13% (se=0.01). Sex-specific analyses found largely overlapping GWAS loci and the genetic correlation (rG) between male and female alcohol consumption was 0.90 (s.e.=0.09, *P*-value=7.16 × 10^−23^). Using LD score regression, genetic overlap was found between alcohol consumption and years of schooling (rG=0.18, s.e.=0.03), high-density lipoprotein cholesterol (rG=0.28, s.e.=0.05), smoking (rG=0.40, s.e.=0.06) and various anthropometric traits (for example, overweight, rG=−0.19, s.e.=0.05). This study replicates the association between alcohol consumption and alcohol metabolizing genes and *KLB*, and identifies novel gene associations that should be the focus of future studies investigating the neurobiology of alcohol consumption.

## Introduction

In 2012, 5.9% of all global deaths were attributable to alcohol and roughly a quarter of all deaths in the 20–39-year-age group.^1^ Over 200 diseases are linked to alcohol consumption and the proportion of the global disease burden, measured in disability-adjusted life years, is over 5%.^1^ Almost a quarter of disability-adjusted life years attributable to alcohol consumption were the result of neuropsychiatric disorders such as alcohol use disorders (AUD) and major depressive disorder.^2^

There is a substantial genetic component to the variation in alcohol consumption, although studies of alcohol use have largely focused on AUD to date. A recent meta-analysis of twin and adoption studies estimated the heritability of AUD to be 0.49 (95% confidence interval 0.43–0.53).^3^ A study of twin pairs with genomic data estimated that the single-nucleotide polymorphism (SNP)-based heritability of AUD was 33% (se=0.12).^4^ Studies of the heritability of *alcohol consumption* are far fewer in number, but a study of 2877 twin pairs estimated the heritability of alcohol consumption to be 0.43 (95% confidence interval 0.31–0.56). Using a sample of unrelated parents, the same authors estimated ~18% of the variance in alcohol consumption is attributable to common SNPs.^5^

Specific genetic variants have been linked to variation in alcohol consumption, the most influential being rs671 in the aldehyde dehydrogenase (*ALDH2*) gene and a cluster of variants spanning the alcohol dehydrogenase genes (*ADH1B*, *ADH1C*, *ADH5*, *ADH6*, *ADH7*) located on chromosome 4q23. The catalytically inactive version of *ALDH2* encoded by the A allele of rs671 leads to slower metabolism of acetaldehyde that causes the alcohol flush reaction. This reaction causes alcohol to be aversive for the A carriers of rs671.^6, 7^ As such, this polymorphism is highly protective against alcoholism in Asian populations,^8^ although it has limited impact in European and African populations where it is often monomorphic.^9^ The rs1229984 in *ADH1B* is also associated with an alcohol-flush reaction in Asian populations and thus protects against high alcohol consumption.^10^ It is also found to associate with drinking pattern phenotypes in European and African populations where the minor allele frequency (MAF) is ~1–4%.^9, 11, 12^ Common SNPs in *ADH7* have been associated with alcohol consumption in an Australian cohort of twins.^13^

Genome-wide association studies (GWASs) of alcohol consumption have found few consistently replicable loci outside of the alcohol metabolizing genes. A GWAS of alcohol consumption comprising 47 501 individuals of European ancestry found rs6943555 in *AUTS2* to be associated with alcohol consumption.^14^ This locus did not replicate in a more recent GWAS of alcohol consumption in >105 000 European individuals. However, a SNP in *KLB* was associated with alcohol consumption in humans and the gene product, β-klotho, was found to regulate alcohol preference in mice.^15^

Alcohol consumption has been linked to psychiatric disorders and other health-related traits, although this has generally been limited to epidemiological observations^16, 17^ or by assessing genetic overlap using twin studies.^18, 19^ Levels of alcohol consumption within a population are strongly linked to cardiovascular disease, liver cirrhosis and cancer.^17^ Furthermore, twin studies have shown that genetic factors overlap with alcoholism and depression,^20^ attention-deficit/hyperactivity disorder and externalizing disorders.^18^ Studies that have assessed the SNP-based genetic overlap between alcohol consumption and other traits have been limited to other substance abuse phenotypes. They have found that the polygenic architecture underlying alcohol consumption is shared with tobacco, caffeine and cannabis use.^21, 22^

In the present study we perform the largest GWAS of self-reported alcohol consumption in 112 117 individuals of European ancestry from the UK Biobank (UKB). We also estimate the SNP-based heritability of alcohol consumption and perform sex-specific analyses to investigate whether the phenotypic differences in alcohol consumption in males and females have a genetic basis. Furthermore, as alcohol consumption is often associated with psychiatric and other diseases and health-related traits, we examined the genetic overlap between alcohol consumption and over 200 disease and behavioural traits. Using a polygenic risk score (PRS) approach we also analysed the amount of variance in self-reported alcohol consumption in a completely independent sample, Generation Scotland: the Scottish Family Health Study (GS) (*N*=19 858).

## Materials and methods

### Samples

#### UK Biobank

UKB is a population-based sample comprising 502 629 individuals resident in the United Kingdom between the age of 40 and 69 years.^23^ During the recruitment period from 2006 to 2010, individuals were recruited from 22 centres across the United Kingdom to reflect a broad socioeconomic demographic and mixture of urban and rural residents. During a self-completed touchscreen interview taken at baseline appointment, participants were asked about their current drinking status (never, previous, current, prefer not to say) and were asked to report their average weekly and monthly alcohol consumption of a range of drink types (red wine, white wine, champagne, spirits, beer/cider, fortified wine). These questions were accompanied by pictures providing an example of a single measure of each drink type. From these measures we derived an average intake of alcohol consumption in units per week. We excluded all former drinkers from the analysis and this left 112 117 individuals with data on both alcohol consumption and genome-wide genotype data. We also repeated our analyses with never drinkers excluded (*N*=108 309) and found the GWAS results to be consistent with the GWAS of the full sample.

#### Generation Scotland: the Scottish family health study

GS is a family- and population-based study comprising 24 096 individuals aged between 18 and 99 years.^24, 25^ Genome-wide genotype data are available for 19 858 individuals.^26^ Alcohol consumption was assessed using a preclinical questionnaire. Participants self-identified as current drinkers, former drinkers or never drinkers. Average consumption was self‐reported units of alcohol consumed in the previous week and these questions were prompted by a table containing the average units contained in a single measure of various drink types to assist participants in the calculation of weekly intake. Any GS individuals or relatives of GS individuals were removed from the UKB sample before GWAS analysis.

This study obtained informed consent from all participants and was conducted under generic approval from the National Health Service National Research Ethics Service (approval letter dated 17 June 2011, Ref 11/NW/0382) and under UK Biobank approval 4844 ‘Stratifying Resilience and Depression Longitudinally’ (principal investigator AMM). All components of GS have received ethical approval from the National Health Service Tayside Committee on Medical Research Ethics (REC Reference Number: 05/S1401/89) and written consent for the use of data was obtained from all participants.

### Genotyping and imputation

#### UK Biobank

The majority of the UKB sample was genotyped using the Affymetrix UK Biobank Axiom array (67%) (Santa Clara, CA, USA) with the rest of the sample genotyped using the Affymetrix UK BiLEVE Axiom array. Quality control, phasing and imputation are described in detail elsewhere (http://biobank.ctsu.ox.ac.uk/crystal/refer.cgi?id=155583), (http://biobank.ctsu.ox.ac.uk/crystal/refer.cgi?id=155580), (http://biobank.ctsu.ox.ac.uk/crystal/refer.cgi?id=157020). Briefly, phasing was performed using a modified version of the SHAPEIT2 algorithm^27^ with a combined panel of the UK10K and 1000 Genomes phase 3 reference panels used for imputation.^28^

Individuals were removed from the present study based on non-British ancestry (within those who self-identified as being British, principal component analysis was used to remove outliers, *n*=32 484), high missingness (*n*=0), relatedness (*n*=7948), quality control failure in UK BiLEVE (*n*=187) and gender mismatch (*n*=0). Relatedness was defined as having a KING-estimated kinship coefficient of >0.0442. For the GWAS analysis we used hard-called genotypes with an imputation info score ⩾0.9, MAF ⩾0.1% and Hardy–Weinberg equilibrium (HWE) *P*-value ⩽1 × 10^−6^ (no. of SNPs=12 489 782). SNPs were then filtered to only include those where >80% of the sample had a hard-called genotype.

#### Generation Scotland

GS samples were genotyped using the Illumina Human OmniExpressExome-8v1.0 Bead Chip and Infinum chemistry and processed using the Illumina Genome Studio Analysis software v2011 (Illumina, San Diego, CA, USA). Quality control was performed to remove SNPs with <98% call rate, individuals with a genotyping rate <98% and SNPs with a HWE *P*-value ⩽1 × 10^−6^ and a MAF ⩾1%. This left 561 125 SNPs available for analyses. More details on blood collection and DNA extraction are provided elsewhere.^25^

### Genome-wide association analyses

In the UKB, quality control was performed on the alcohol consumption phenotype to remove extreme values. Weekly intake values of >5 s.d. from the mean were set to missing. For males this was for values >102 units per week and for females for values >89 units per week. Alcohol consumption was then log (units+1) transformed and, as the mean alcohol intake in males was significantly higher than in females, we regressed age and weight in kg onto weekly units of alcohol consumed in males and females separately. We then took the residuals from these regressions and pooled the male and female residuals together to create the alcohol consumption phenotype. A male-only and female-only GWAS was also performed.

GWAS was performed using PLINK v1.9 (ref. 29) testing for associations between SNPs and alcohol consumption in unrelated individuals with location of UKB assessment centre, genotyping batch and 15 principal components included as covariates. In order to distinguish independent GWAS signals, SNPs with an association *P*-value ⩽1 × 10^−4^ were subjected to linkage disequilibrium (LD)-based clumping that was performed in PLINK,^29^ using an LD *r*^2^ cutoff of 0.2 and a 500 kb sliding window. Conditional SNP analyses were performed using PLINK for the 8 SNPs on chromosome 4q found to be associated with alcohol consumption. Each SNP was retested for association using the same method as described for the GWAS but using one of the seven other SNPs as a covariate. Conditional SNP analyses were also performed in GCTA using a stepwise model selection procedure to confirm which SNPs were independently associated.^30, 31^

### Heritability and genetic correlation analyses

Univariate GCTA-GREML analyses were performed to estimate the SNP-based heritability of alcohol consumption in the whole UKB sample and then for males and females separately. A genetic relationship matrix was created for an unrelated subsample using a cutoff of 0.025 (*N*=89 175) and used to estimate heritability.^31, 32, 33^ The genetic correlation between male and female alcohol consumption was estimated using LD score regression following the pipeline designed by Bulik-Sullivan *et al.*^34^ This method exploits the correlational structure of SNPs across the genome and uses test statistics provided from GWAS summary estimates to calculate the genetic correlations between traits.^35^ False discovery rate was used to correct *P*-values for multiple testing. We supplied the GWAS summary statistics from the GWAS of male and female alcohol consumption. Using our GWAS summary statistics, the genetic overlap between alcohol consumption and over 200 other disease traits was assessed using LD score regression^34^ implemented in the online software LD Hub (http://ldsc.broadinstitute.org/).^36^

### PRS analyses

PRSs were created in GS using raw genotype data using the software PRsice^37^ using the GWAS summary statistics from the UKB GWAS of alcohol consumption. PRSs were created using *P*-value thresholds ranging from 0.01 to 0.5 in increments of 0.01 using LD pruning parameters of *r*^2^=0.1 over 250 kb windows. The PRS *P*-value threshold found to explain most of the variance in alcohol consumption in GS was at 0.42 and hence this PRS was used to test for association in subsequent analyses. Association between alcohol PRS and traits of interest was performed in AS-Reml-R and an inverse relationship matrix created from the pedigree information in GS was used to control for relatedness in the sample. Four principal components were fit as fixed-effect covariates to control for population stratification. All traits and PRS were scaled to have a mean of 0 and a s.d. of 1 such that the βs reported are standardized. The variance explained by PRS was calculated by multiplying the PRS by its regression coefficient. This value was divided by the variance of the phenotype analysed to give a coefficient of determination between 0 and 1.^38^ As 5 traits were tested for their association with alcohol consumption PRS, Bonferroni correction for PRS analyses required a *P*-value of 0.01 (0.05/5) as the threshold for statistical significance.

### The eQTLs and gene-based analyses

SNPs that were significantly associated with alcohol consumption (*P*<5 × 10^−8^) in the GWAS were assessed to determine whether they were potential expression quantitative trait loci (eQTLs) using the Genotype Tissue Expression Portal (GTEx) (http://www.gtexportal.org). GTEx uses gene expression data from multiple human tissues linked to genotype data to provide information on eQTLs. Gene-based analyses were performed using MAGMA,^39^ derived from SNP summary data from the GWAS of alcohol consumption in this sample. The 1000 genomes European reference panel (phase 1, release 3) was used to account for LD in the sample. SNPs were mapped to genes using the NCBI 37.3 gene locations and Entrez gene IDs. This resulted in 18 024 genes available for analysis that, after Bonferroni correction (0.05/18 024), gave a threshold for statistical significance at 2.8 × 10^−6^.

## Results

The mean self-reported alcohol consumption in the 112 117 UKB individuals who contributed to the analysis in the present study was 15.13 units per week (s.d.=16.56). The mean age of participants was 59.6 (s.d.=7.95). The sample comprised 59 088 females (52.7%). Females had a significantly lower mean weekly alcohol intake compared with males (10.03 (s.d.=11.83) vs 20.81 (s.d.=19.04) units per week), *P*⩽2 × 10^−16^.

The SNP-based heritability of alcohol consumption in the total sample was estimated to be 0.13 (s.e.=0.006, *P*=1.3 × 10^−119^). The SNP heritability in males was estimated to be 0.15 (s.e.=0.01, *P*=2.3 × 10^−41^) and in females 0.13 (s.e.=0.01, *P*=8.1 × 10^−36^). The SNP heritability in males was not significantly higher than in females (*Z*-score=1.43, *P*-value=0.152). The SNP-based genetic correlation between male and female alcohol consumption using LD score regression was 0.9 (s.e.=0.09, *P*-value=7.16 × 10^−23^), suggesting that the genetic factors influencing alcohol consumption in males and females in this sample were largely, but not completely, overlapping.

**Figure 1:**
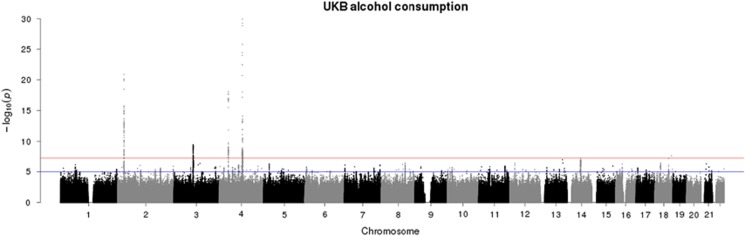
Manhattan plot of genome-wide association study (GWAS) of alcohol consumption in UK Biobank (UKB; *N*=112 177). Red line indicates threshold for genome-wide significance (*P*⩽5 × 10^−8^) and the blue line for suggestive significance (*P*⩽5 × 10^−6^).

**Figure 2:**
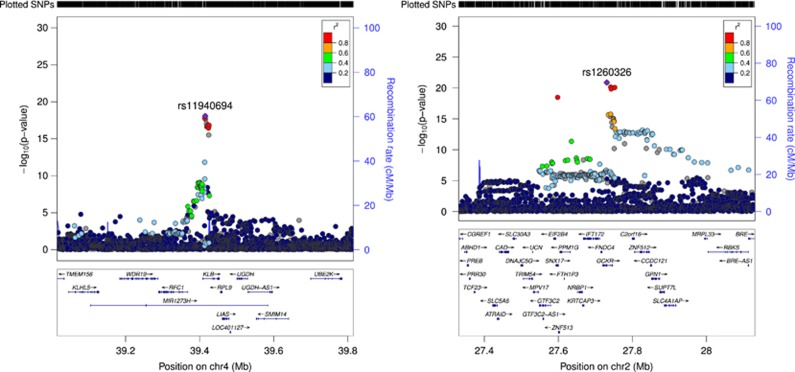
Linkage disequilibrium (LD) zoom plots of 2 common single-nucleotide polymorphisms (SNPs; minor allele frequency (MAF) >5%) most significantly associated (*P*<5 × 10^−8^) with alcohol consumption in UK Biobank. (**a**) The rs2872821 on chromosome 4 in *KLB* and (**b**) rs1260326 on chromosome 2 located in *GKCR* (http://locuszoom.sph.umich.edu/).

The genome-wide association statistics deviated slightly from the null (λ_GC_=1.092) (Supplementary Figure 1), though the LD score regression intercept of 1.001 suggested that the inflation of the test statistic was due to polygenicity rather than any population stratification.^35^ Genome-wide significant associations (*P*<5 × 10^−8^) were obtained for 14 loci after clump-based pruning (Table 1 and Figure 1). These included 8 hits on chromosome 4q23/4q22.3 that span ~2.3 MB and several alcohol dehydrogenase genes (*ADH1B*, *ADH1C*, *ADH5*) that have all previously been implicated in alcohol consumption.^9, 12^ Conditional SNP GWAS of the variants on chromosome 4q suggests that there are 4 independent hits in this region: rs145452708 in the *ADH1B/ADH1C* region, rs29001570 in *ADH5*, rs35081954 in *ADH1C* and rs193099203 that lies in an intergenic region of 4q23. Most of the associated variants on 4q23 are relatively rate (MAF 0.006–0.01) with the minor allele associated with lower alcohol consumption. However, rs35081954 is a common insertion/deletion polymorphism with a MAF of 0.42. Two hits were identified on chromosome 4p14 in the *KLB* gene that was previously associated with alcohol consumption in a large GWAS of >105 000 Europeans.^15^ Conditional SNP analyses suggested these two SNPs were not completely independent and identified rs11940694 as the lead SNP associated with alcohol consumption (Supplementary Table 1). Novel hits included a SNP on chromosome 2p23.3 in the *GCKR* gene. The SNPs in *KLB* and *GCKR* were the most strongly associated common (MAF >5%) SNPs with MAFs of >0.28. Regional association plots for the common SNPs in *KLB* and *GCKR* are shown in Figure 2. Additional novel hits on chromosome 3p12.1 (*CADM2*) and chromosome 18q22.3 (*FAM69C*) were also identified (Table 1). Two SNPs in *CADM2* were associated at genome-wide significance level but conditional SNP analyses found rs9841829 to be the lead SNP in this region. These loci will be reviewed in greater detail in the discussion. Regional association plots for all SNPs presented in Table 1 are shown in Supplementary Figures 2.

A GWAS of alcohol consumption was performed excluding individuals who were identified as never drinkers. The genetic regions associated with alcohol consumption in current drinkers overlapped with those reported in the total sample with the exception of rs8012947 in the *AT-rich interactive domain 4A* (*ARID4A)* gene on chromosome 14 (Supplementary Table 2 and Supplementary Figure 11).

Sex-specific GWASs of alcohol consumption were also performed (Supplementary Figures 12 and 13). Only one novel locus (rs140089781) was identified in males on chromosome 2 in catenin-α2 (*CTNNA2*). rs140089781 was not analysed in females because of the low MAF of this SNP (MAF <0.001). All other loci identified in males or females had previously been identified at the level of genome-wide significance in the total sample (Supplementary Table 3).

The gene-based analyses found 41 genes to be significantly associated with alcohol consumption (Supplementary Table 4). The top hits were genes identified in the single SNP analyses (*KLB*, *CADM2*, *GCKR*) and genes in the 4q region were also found to be significantly associated with alcohol consumption at the gene level (*ADH1C*, *C4orf17*). Among the 41 loci associated at the gene level, genes of interest include the *dopamine receptor D2* (*DRD2*) gene, previously associated with addiction phenotypes,^40^ *chromodomain Y-like* (*CDYL*) protein previously associated with alcohol consumption, cAMP-specific 3′,5′-cyclic *phosphodiesterase 4B* (*PDE4B*) associated with alcohol preference in rodents,^41, 42^ *zinc-finger protein 512* (*ZNF512*) associated with oral cavity cancer^43^ and *protein phosphatase 1G* (*PPM1G*) found to be hypermethylated in individuals with AUD.^44^

Four SNPs located across *KLB*, *CADM2* and *GCKR* were found to be eQTLs according to the GTEx database (Supplementary Table 5). Notably, rs11940694 in *KLB* was found to be an expression QTL for *RCF1* and *RPL9* in the cerebellum. rs9841829 in *CADM2* is associated with expression of *CADM2* levels in lung and adipose tissue.

**Figure 3:**
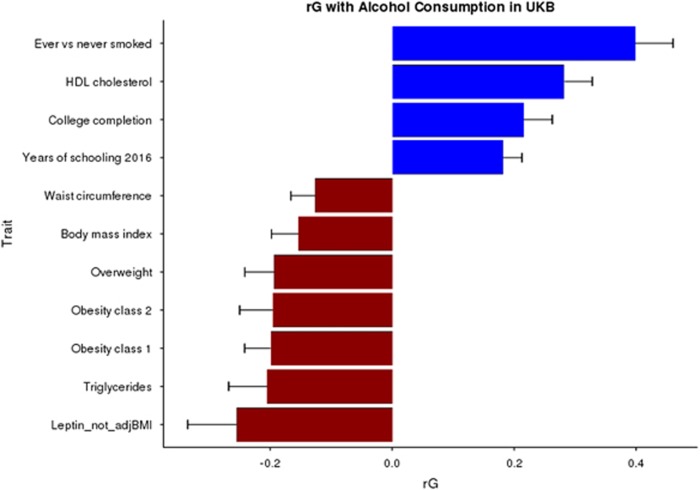
Genetic correlation between Alcohol Consumption in UK Biobank (UKB) and other traits using linkage disequilibrium (LD) score regression implemented in LD Hub. All traits presented pass false discovery rate (FDR) correction for multiple testing. HDL, high-density lipoprotein.

The genetic correlations between alcohol consumption and 212 other health and behavioural traits were calculated using GWAS summary statistics and LD score regression^34^ implemented in the online software LD Hub.^36^ After correction for multiple testing, 11 traits had a significant genetic correlation (*P*<0.05) with alcohol consumption (Figure 3). Smoking status had the strongest positive genetic correlation (rG) with alcohol consumption (rG=0.40, s.e.=0.06, *P*=1.4 × 10^−10^) followed by high-density lipoprotein (HDL) cholesterol levels (rG=0.28, s.e.=0.05, *P*=6.9 × 10^−10^). Significant negative genetic correlations were observed for overweight (rG=−0.19, s.e.=0.05, *P*=5.8 × 10^−5^) and a range of other anthropometric traits pertaining to body mass index (BMI) and obesity, such as obesity class 2 (rG=−0.20, s.e.=0.06, *P*=3 × 10^−4^) that categorizes severely obese individuals with a BMI ranging from 35.0 to 39.9 kg/m^2^.

Genetic correlations were also calculated using the sex-specific GWAS summary statistics (Supplementary Figures 14 and 15). Male alcohol consumption showed a negative genetic correlation with childhood height (rG=−0.23, s.e.=0.07, *P*=0.002), childhood obesity (rG=−0.26, s.e.=0.08, *P*=0.001) and infant head circumference (rG=−0.34, s.e.=0.11, *P*=0.002). Female alcohol consumption was genetically correlated with college completion (rG=0.3, s.e.=0.06, *P*=1.4 × 10^−7^), height (rG=0.18, s.e.=0.04, *P*=2.4 × 10^−5^) and bipolar disorder (rG=0.22, s.e.=0.08, *P*=0.005). Several metabolic traits were found to have negative genetic correlations with female alcohol consumption including low-density lipoprotein cholesterol (rG=−0.23, s.e.=0.07, *P*=9 × 10^−4^) and leptin (rG=−0.41, s.e.=0.11, *P*=3 × 10^−4^).

Using the UKB GWAS summary statistics PRS for alcohol consumption were calculated in GS to determine the amount of variance that could be explained using a risk-score approach. PRSs for alcohol consumption in GS were found to be positively associated with alcohol consumption (β=0.08, *P*=6.5 × 10^−24^) but only 0.6% of the variance in alcohol consumption was explained (Table 2). PRSs were also tested for association with traits found to have a significant genetic correlation with alcohol consumption in the LD score regression analyses. BMI, weight, hip circumference, smoking status and HDL cholesterol levels were all available in the GS cohort. Significant associations between alcohol PRS and smoking were detected after adjustment for multiple testing (β=−0.143, *P*=0.01), and with HDL cholesterol (β=0.03, *P*=4.3 × 10^−5^) and BMI (β=0.02, *P*=0.0013) (Table 2). No significant associations were found between PRSs and hip circumference.

## Discussion

In the present study we identify eight independent loci associated with alcohol consumption. Four of these loci are located among a cluster of alcohol metabolism genes on chromosome 4q23 (*ADH1B/ADH1C* and *ADH5*) and have been previously identified as risk loci for alcohol-related phenotypes.^9, 12^ A SNP in *KLB* was also associated with alcohol consumption and this locus has previously been identified in a large meta-analysis of alcohol consumption in Europeans.^15^ The remaining three hits are novel loci (*GCKR, CADM2* and *FAM69C*) in the context of alcohol consumption and as such this study presents a novel contribution to the genetics of alcohol consumption.

Identifying the causal variants located on 4q23 will prove to be challenging because of the low MAF of the variants associated with alcohol consumption in this region. Previous studies have shown that rs1229984 in *ADH1B*, which is associated with rapid alcohol metabolism, is strongly protective against high alcohol consumption.^10^ It is possible that the low-frequency variants identified in 4q23 are tagging rs1229984. However, rs1229984 deviated from HWE in the UKB (HWE *P*=1.5 × 10^−78^) and therefore was not analysed as part of the GWAS. The rs29001570 in *ADH5* clearly represents an independent locus as the *r*^2^ with the other SNPs in 4q23 is <0.01. We believe this is the first GWAS of alcohol consumption to detect genome-wide significant association with *ADH5*, although a GWAS of alcohol dependence found suggestive evidence of association with *PDLIM5* that is adjacent to *ADH5*.^12^ ADH5 is a formaldehyde dehydrogenase with low affinity for alcohol and therefore its role in alcohol metabolism and consumption is as yet unknown. rs145452708 that is located in the region between *ADH1B/ADH1C* and rs35081954 is a common variant located in intron 1 of *ADH1C.* ADH1B and ADH1C are alcohol dehydrogenases expressed in the liver and are primarily responsible for the catabolism of ethanol. Variants in *ADH1B* and *ADH1C* have previously been associated with various alcohol consumption phenotypes.^9, 12, 45^

The gene encoding β-Klotho (*KLB)* has recently been associated with alcohol consumption in a large meta-analysis of >105 000 Europeans. Schumann *et al.*^15^ found that brain-specific *klb-*knockout mice have increased alcohol preference. They also found that β-Klotho is a receptor for the liver-expressed hormone FGF21 that acts on the brain and inhibits alcohol consumption in mice. We are not aware of any sample overlap between UKB and the sample used in the study by Schumann *et al.*^15^. GS individuals were present in the GWAS reported by Schumann *et al.*^15^ but GS individuals identified in the UKB sample were removed before GWAS analysis. The study by Schumann *et al.*^15^ identified rs11940694 to be associated with alcohol consumption and this is the same SNP we report as associated in this sample. We therefore provide evidence of replication of the association of this variant with alcohol consumption. An additional SNP (rs9991733) in *KLB* was associated with alcohol consumption in UKB; however, the minor allele of this SNP conferred risk for high consumption rather than protection. This SNP is in low LD with rs11940694 (*r*^2^=0.21, *D*’=0.87); however, conditional SNP analyses showed that these associations were not completely independent.

Novel loci found to be associated with alcohol consumption in this study include *GCKR*, *CADM2* and *FAM69C. GCKR* encodes the glucokinase regulatory protein that is produced by hepatocytes and is responsible for phosphorylation of glucose in the liver. rs1260326 in *GCKR* is a coding missense SNP and this variant has been associated with over 25 metabolic traits including type II diabetes, fasting insulin levels and total cholesterol levels.^46^ *CADM2* is a brain expressed gene encoding cell adhesion molecule 2 that has previously been associated with processing speed and educational attainment in the UKB sample and processing speed in the CHARGE consortium cohort.^47, 48^ A recent study of reproductive success and risk-taking propensity also found genome-wide significant associations with SNPs in *CADM2*.^49^ *CADM2* has also been associated with lifetime cannabis use in a GWAS of ~32 000 individuals.^50^

Several additional genes associated with alcohol consumption were identified through MAGMA gene-based analyses, including the gene *CDYL*. *CDYL* was also associated with alcohol consumption at the gene level and a SNP in this gene has previously shown some association with alcohol consumption in a GWAS of 47 501 Europeans.^14^ *PDE4B* was associated at the gene level and encodes a protein that regulates alcohol-induced neuroinflammation in the central nervous system.^51^ Animal models have shown that inhibition of PDE4B leads to reduced alcohol intake in both mice and rats.^41, 42^ A GWAS found *ZNF512* to be associated with oral cavity cancer.^43^ The gene-based analysis in the present study found this gene to be associated with alcohol consumption. This is notable considering that alcohol consumption is a leading cause of oral cavity cancer, suggesting that the link between *ZNF512* and alcohol consumption may underlie its association with oral cancer.

Using LD score regression, a significant positive genetic correlation between alcohol consumption and smoking was identified. The overlap between alcohol consumption and smoking is well documented and other studies have shown polygenic overlap between weekly alcohol intake and the number of cigarettes smoked (rG=0.44).^21^ A positive genetic correlation between HDL cholesterol and alcohol consumption was also found. Increased alcohol consumption is associated with increased HDL levels^52^ and a Mendelian randomization study of alcohol consumption and lipid profiles found a causal effect of alcohol on increased HDL levels in the low to moderate intake range.^53^ rs1260326 in *GCKR* has previously been associated with HDL levels^54^ and this was one of the most significant associations with alcohol consumption in the present study. Furthermore, alcohol PRSs in GS were nominally associated with increased HDL. Our findings provide further support for the positive relationship between alcohol and HDL levels.

A positive genetic correlation was observed between alcohol consumption and years of schooling and college completion. Other studies have found an association with educational attainment and alcohol consumption. A study of individuals in the British Cohort Study found that educational attainment was linked to increased risk of daily alcohol consumption at age 34 years and that this was stronger in females.^55^ We also find a stronger genetic correlation with college completion in females compared with males (rG 0.33 vs 0.10). Another study on the British Cohort Study found mental ability at age 10 years was linked to increased alcohol consumption and alcohol problems at age 30 years.^56^ Childhood academic ability at age 11 years has been positively correlated with alcohol consumption in early adolescence.^57^ This relationship extends into older age as a link between alcohol consumption and cognitive ability in 70 year olds was observed in the Lothian Birth Cohort, although after correcting for socioeconomic status and childhood intelligence much of this association was attenuated.^58^

Negative genetic correlations were observed for several anthropometric traits including BMI and obesity, such that genetic variants that increased risk for alcohol consumption decrease the risk for overweight phenotypes. As alcohol has a caloric content of 7.1 kcal g^−1^, it seems counterintuitive that increased alcohol consumption negatively correlates with measures of obesity. A genetic variant in *FTO* that increases risk for obesity is also associated with increased risk for alcohol dependence.^59^ However, there is some evidence that high alcohol consumers have altered metabolism. A study of alcohol-dependent individuals found that those consuming the highest levels of alcohol had increased metabolism and decreased fat mass and leptin levels.^60^ Furthermore, high alcohol consumption can impair nutrient absorption.^61^ The many studies of obesity and alcohol consumption have indicated a complex relationship^62^ with reports of both negative and positive correlations between alcohol and BMI.^63, 64^ We also report a negative genetic correlation between alcohol consumption and leptin levels (rG=−0.26, s.e.=0.08). Leptin is a hormone generated by adipose cells and is involved in inducing satiety. Moderate alcohol consumption has been linked to inhibition of leptin in healthy volunteers^65^ but a study of healthy postmenopausal women found the ingestion of 15–30 g of alcohol per day associated with increased serum leptin levels.^66^ Work in rodents has shown that alcohol consumption decreases serum leptin but increases leptin levels in adipose tissue.^67^ These findings are intriguing and the genetic link between hunger, obesity and alcohol consumption warrants further investigation.

The traits that show a genetic correlation with alcohol consumption may correlate because of shared genetic effects on both traits or because of causal pathways between traits. In addition, genetic confounding may influence the correlations. A genetic correlation between smoking and alcohol consumption was identified in this study. As smoking also influences triglyceride levels^68^ this may be an indirect cause of the negative genetic correlation between alcohol consumption and triglyceride levels. Future studies should use Mendelian randomization analyses with the genetic variants identified in this study to probe causal relationships between alcohol use and health traits. Assortative mating may also influence the genetic correlations identified in this study. Alcohol consumption and nicotine use have been shown to be factors for nonrandom mating,^69^ and it is unclear what effect, if any, this would have on the genetic correlations presented in this study.

This investigation has several advantages compared with previous studies. We studied a single large sample of ancestrally similar individuals from a relatively narrow age range, all of whom were residing in the United Kingdom at the time of interview. Using a sample where all individuals are exposed to similar social norms is advantageous as cultural influences are an important influence on alcohol intake patterns.^70^ Furthermore, all data were subject to the same quality control procedures and these factors would have contributed to our ability to detect eight independent genome-wide significant loci when similar-sized alcohol consumption GWAS did not.^15^ The main limitation of this study is the reliance on self-reported alcohol consumption and the reliability of this measure. However, the UKB touchscreen interview used to ascertain alcohol intake used pictures to represent different drink sizes that may have increased reliability. Finally, the results presented here may only be applicable to middle- to older-aged white British individuals. Considering that genetic influences on alcohol consumption change across the life course,^71^ the variants identified in this study may have limited relevance outside of this demographic. It is important to note that the mean weekly alcohol consumption for males in this sample was 20.8 units per week, almost 6 units higher than the UK Chief Medical Officers’ recommendation of 14 units per week for men and women. This suggests that among this demographic, males are regularly consuming levels of alcohol that could cause risks to health.

This study presents the largest single GWAS of alcohol consumption to date and identifies eight genetic loci, at least three of which are novel. The SNP heritability of alcohol consumption is described for the first time with 13% of the variance in alcohol consumption attributable to genetic factors in this sample. Many of the genes identified in this study are expressed in the liver, suggesting that alcohol metabolism is an important driver of differences in consumption. Genetic correlation analyses found associations between alcohol consumption and many positive health and behavioural traits such as higher education, lower obesity and high HDL cholesterol levels. Although it is clear that high levels of alcohol consumption are linked to poorer health outcomes, the positive genetic correlations identified in this study are intriguing and warrant further exploration. Future work should focus on characterizing the biological role of the newly identified genetic variants in alcohol consumption and the potential causal relationships between alcohol use and health-related traits.

## Figures and Tables

**Table 1:** Fourteen loci reaching genome-wide significance for association with alcohol consumption in UKB after performing clump-based LD pruning

*SNP*	*CHR*	*POS*	*A1/A2*	*Freq*	*β (s.e.)*	P	*Genes*
**rs145452708**	4	100248642	C/G	0.01	−0.034 (0.003)	1.15 × 10^−30^	*ADH1B/ADH1C*
**rs193099203**	4	99630017	T/C	0.007	−0.031 (0.003)	3.79 × 10^−25^	—
**rs1260326**	2	27730940	T/C	0.39	−0.028 (0.003)	1.34 × 10^−21^	*GCKR*
**rs11940694**	4	39414993	A/G	0.39	−0.027 (0.003)	8.4 × 10^−19^	*KLB*
**rs29001570**	4	99994405	C/T	0.006	−0.026 (0.003)	9.58 × 10^−19^	*ADH5*
rs3114045	4	100252560	T/C	0.13	−0.020 (0.003)	7.98 × 10^−12^	*ADH1B/ADH1C*
rs140280172	4	100832564	A/C	0.005	−0.020 (0.003)	2.89 × 10^−11^	*DNAJB14*
**rs9841829**	3	85569361	G/T	0.23	0.019 (0.003)	3.36 × 10^−10^	*CADM2*
**rs35081954**	4	100270818	CTG/C	0.42	0.018 (0.003)	2.14 × 10^−9^	*ADH1C*
rs375507729	3	85062293	T/TAA	0.44	0.018 (0.003)	3.19 × 10^−9^	*CADM2*
rs9991733	4	39420994	G/A	0.28	0.018 (0.003)	3.80 × 10^−9^	*KLB*
rs149127347	4	99101007	G/T	0.003	−0.018 (0.003)	4.42 × 10^−9^	—
rs145329623	4	98528709	G/A	0.003	−0.017 (0.003)	5.68 × 10^−9^	*STPG2*
**18:72124965**	18	72124965	CCTCCCG/C	0.05	0.017 (0.003)	2.18 × 10^−8^	*FAM69C*

Abbreviations: CHR, chromosome; Freq, A1 frequency; GWAS, genome-wide association study; LD, linkage disequilibrium; POS, base pair position; SNP, single-nucleotide polymorphism; UKB, UK Biobank.

Genes are reported if SNPs located ±10 kb of the locus. SNPs highlighted as bold represent completely independent associations identified by conditional SNP analyses.

**Table 2:** PRS analyses: PRS for alcohol consumption in Generation Scotland created using summary statistics of alcohol consumption GWAS in UKB

*Trait (n)*	*β (s.e.)*	*Z ratio*	*r*^2^	P*-value*
Units per week (17 461)	0.080 (0.007)	10.10	0.6%	**6.5 × 10**^**−24**^
Ever smoke (19 289)	−0.143 (0.004)	−0.009	0.04%	**0.01**
BMI (19 771)	−0.024(0.007)	−3.21	0.056%	**0.0013**
Hips (1958)	−0.009 (0.008)	−1.24	0.009%	0.21
HDL cholesterol (19 108)	0.03 (0.007)	4.09	0.03%	**0.000043**

Abbreviations: BMI, body mass index; CHR, chromosome; Freq, A1 frequency; GWAS, genome-wide association study; HDL, high-density lipoprotein; PRS, polygenic risk score; POS, base pair position; SNP, single-nucleotide polymorphism; UKB, UK Biobank.

Association analyses performed in AS-Reml-R using pedigree information to control for relatedness. Units per week phenotype for current drinkers only. All models adjusted for age, sex and four multidimensional scaling (MDS) components to control for population stratification. Bold highlighted *P*-values are statistically significant after correction for multiple testing (*P*⩽0.01).
