# Supplementary material for: Genome-wide association study of alcohol consumption and genetic overlap with other health-related traits in UK Biobank (N=112 117)
Source: Mol Psychiatry. 2017 Jul 25;22(10):1376–84. doi: 10.1038/mp.2017.153 (PMC5622124; doi:10.1038/mp.2017.153)
Supplement: Supplementary Information [file mp2017153x1.docx]

**Supplemental material for ‘GWAS of alcohol consumption and genetic overlap with other health related traits’ Clarke et al.**

Contents

[Supplemental Figure 1: QQ plot of alcohol consumption GWAS in UKB. 3](#_Toc480974155)

[Supplementary Figure 2: Locus Zoom plot of rs145452708 and LD with SNPs in the 400kb surrounding region. 4](#_Toc480974156)

[Supplementary Figure 3: Locus Zoom plot of rs193099203 and LD with SNPs in the 400kb surrounding region. 5](#_Toc480974157)

[Supplementary Figure 4: Locus Zoom plot of rs29001570 and LD with SNPs in the 400kb surrounding region. 6](#_Toc480974158)

[Supplementary Figure 5: Locus Zoom plot of rs3114045 and LD with SNPs in the 400kb surrounding region. 7](#_Toc480974159)

[Supplementary Figure 6: Locus Zoom plot of rs140280172 and LD with SNPs in the 400kb surrounding region. 8](#_Toc480974160)

[Supplementary Figure 7: Locus Zoom plot of rs9841829 and LD with SNPs in the 400kb surrounding region. 9](#_Toc480974161)

[Supplementary Figure 8: Locus Zoom plot of rs9991733 and LD with SNPs in the 400kb surrounding region. 10](#_Toc480974162)

[Supplementary Figure 9: Locus Zoom plot of rs149127347 and LD with SNPs in the 400kb surrounding region. 11](#_Toc480974163)

[Supplementary Figure 10: Locus Zoom plot of rs145329623 and LD with SNPs in the 400kb surrounding region. 12](#_Toc480974164)

[Supplementary Figure 11: Manhattan plot of alcohol consumption in current drinkers only in UKB (N=108,309). 13](#_Toc480974165)

[Supplementary Figure 12: Manhattan plot of alcohol consumption in females in UKB (N=59,088). 14](#_Toc480974166)

[Supplementary Figure 13: Manhattan plot of alcohol consumption in males in UKB (N=53,089). 15](#_Toc480974167)

[Supplementary Figure 14: Genetic correlation between female alcohol consumption in UKB and other traits using LD score regression implemented in LDHub. 16](#_Toc480974168)

[Supplementary Figure 15: Genetic correlation between male alcohol consumption in UKB and other traits using LD score regression implemented in LDHub. 17](#_Toc480974169)

[Supplementary Table 1: Conditional GWAS analyses of SNPs associated with alcohol consumption on chromosome 4q. 18](#_Toc480974170)

[Supplementary Table 2: Thirteen loci reaching genome-wide significance for association with alcohol consumption in current drinkers (N=108,309) in UKB. 19](#_Toc480974171)

[Supplementary Table 3: Loci reaching genome-wide significance for association with alcohol consumption in UKB in males and females only. 20](#_Toc480974172)

[Supplementary Table 4: Results of MAGMA gene-based association analyses. 22](#_Toc480974173)

[Supplementary Table 5: GTEx analysis of SNPs associated with alcohol consumption 23](#_Toc480974174)


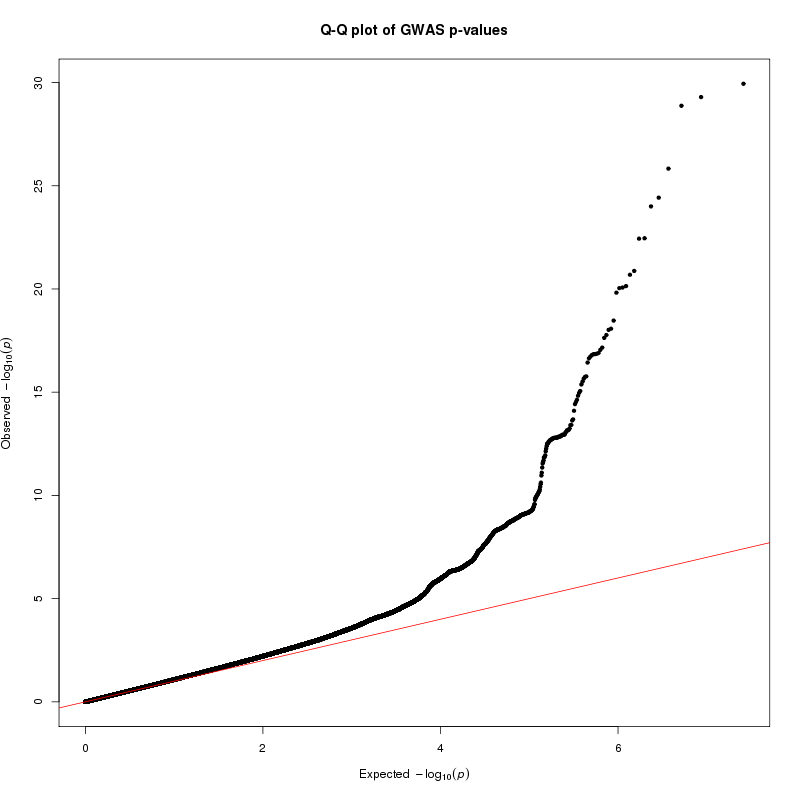


Supplemental Figure 1: QQ plot of alcohol consumption GWAS in UKB. N=112,177 individuals, NSNPS = 12,489,781. λGC=1.092, λ1000=1.0008, LD regression intercept =1.0123 (0.008)


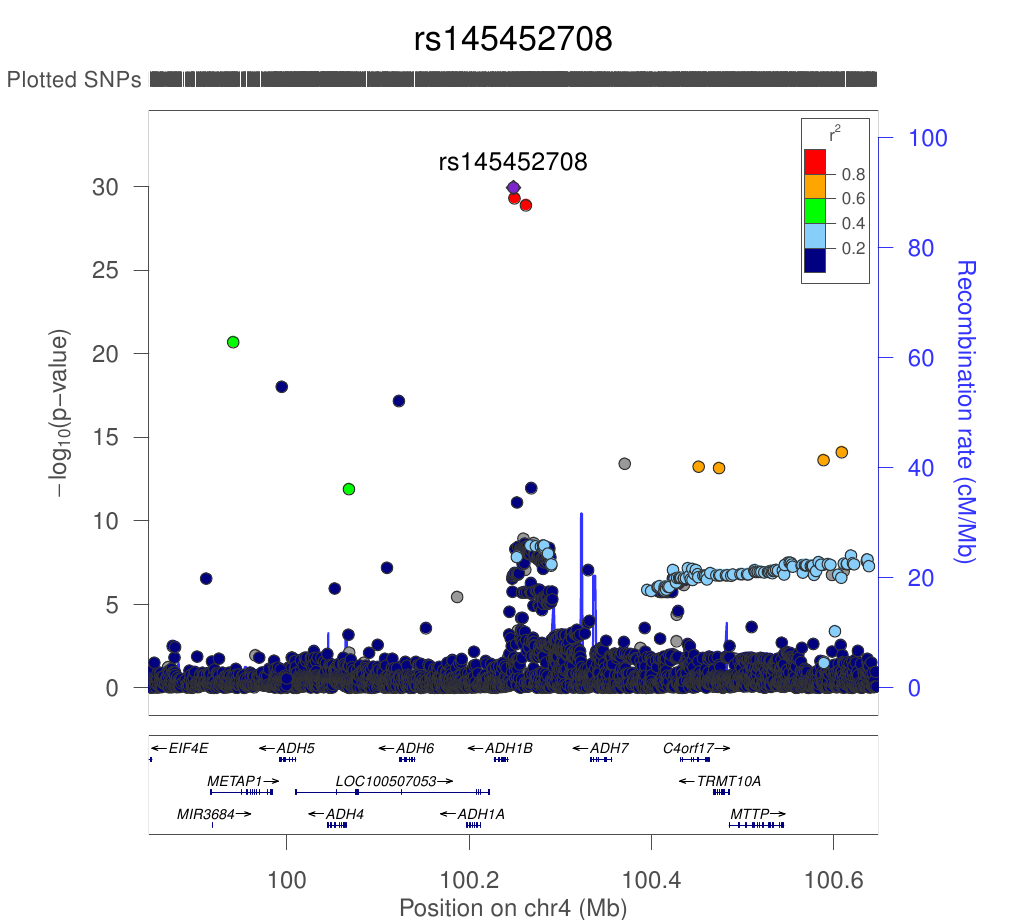


# Supplementary Figure 2: Locus Zoom plot of rs145452708 and LD with SNPs in the 400kb surrounding region.


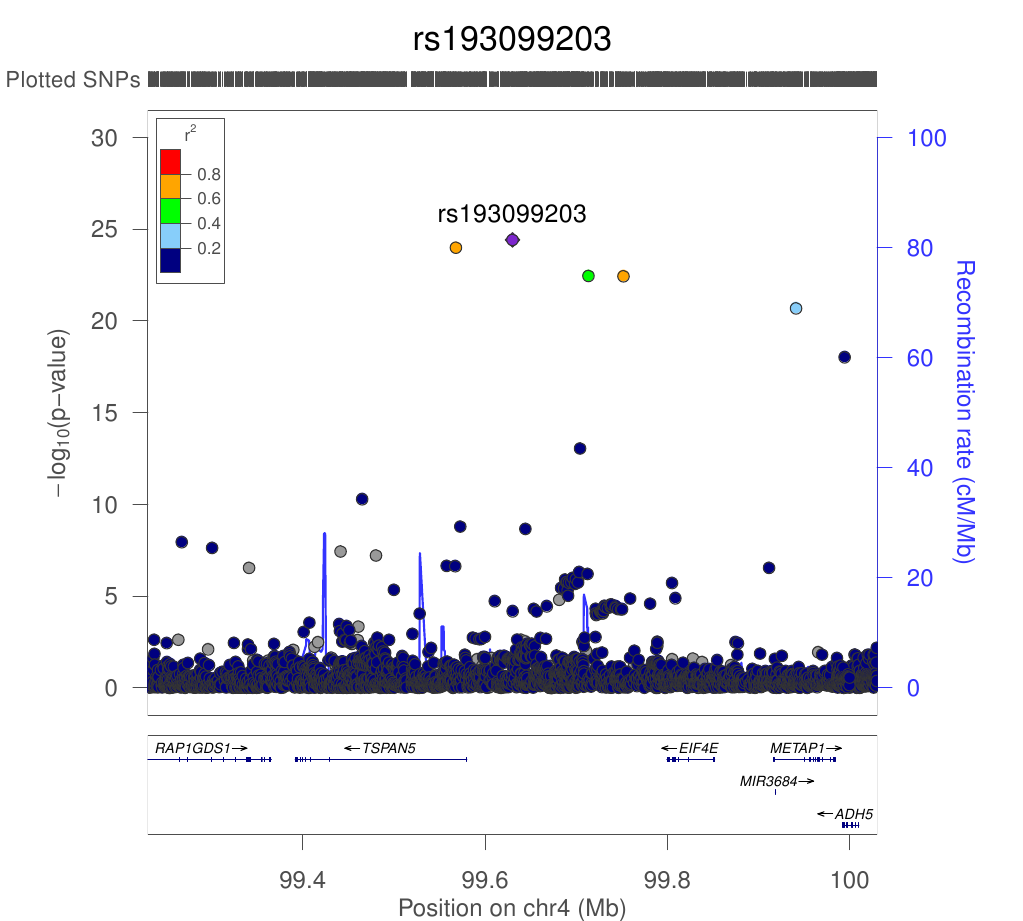


# Supplementary Figure 3: Locus Zoom plot of rs193099203 and LD with SNPs in the 400kb surrounding region.


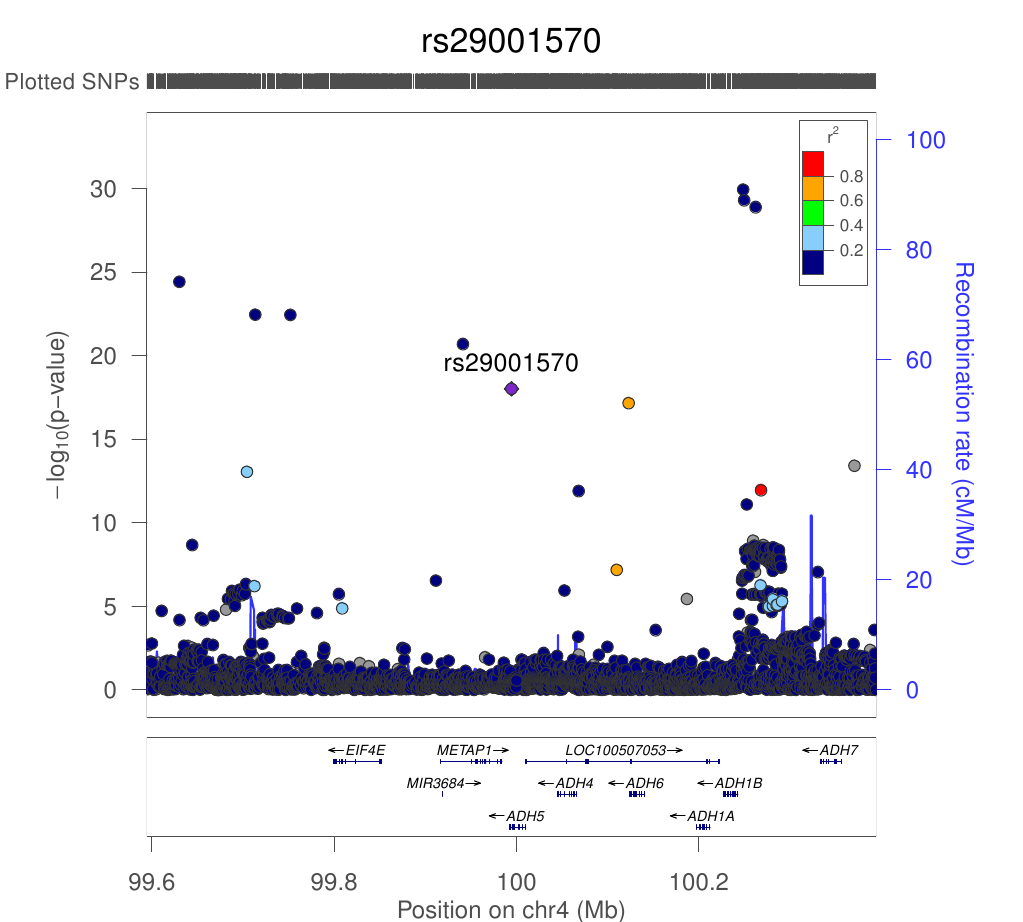


# Supplementary Figure 4: Locus Zoom plot of rs29001570 and LD with SNPs in the 400kb surrounding region.


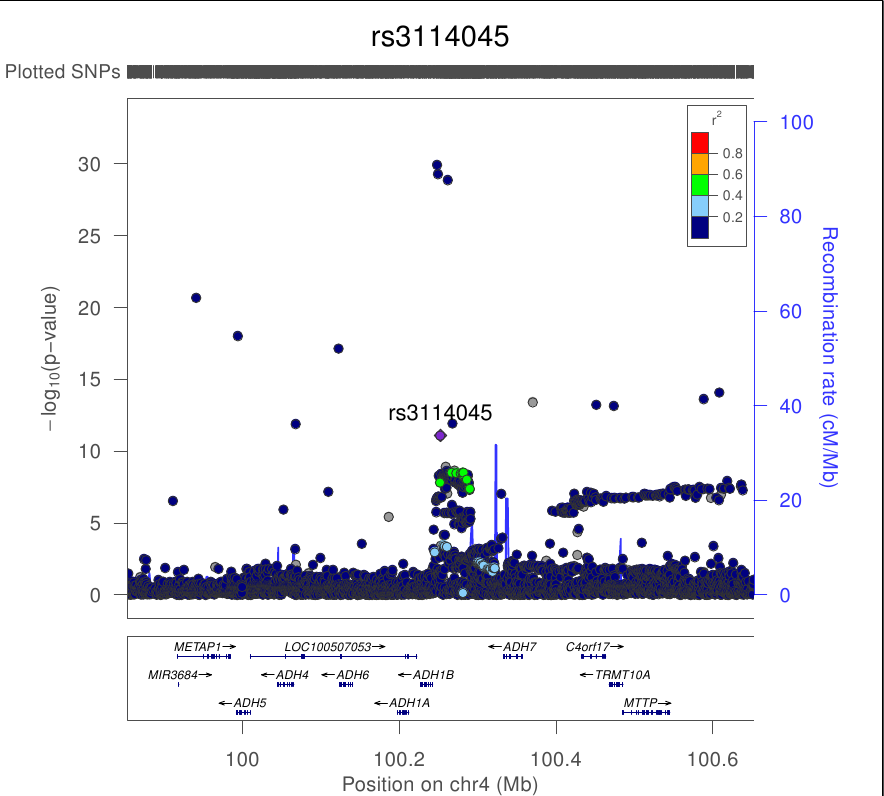


# Supplementary Figure 5: Locus Zoom plot of rs3114045 and LD with SNPs in the 400kb surrounding region.


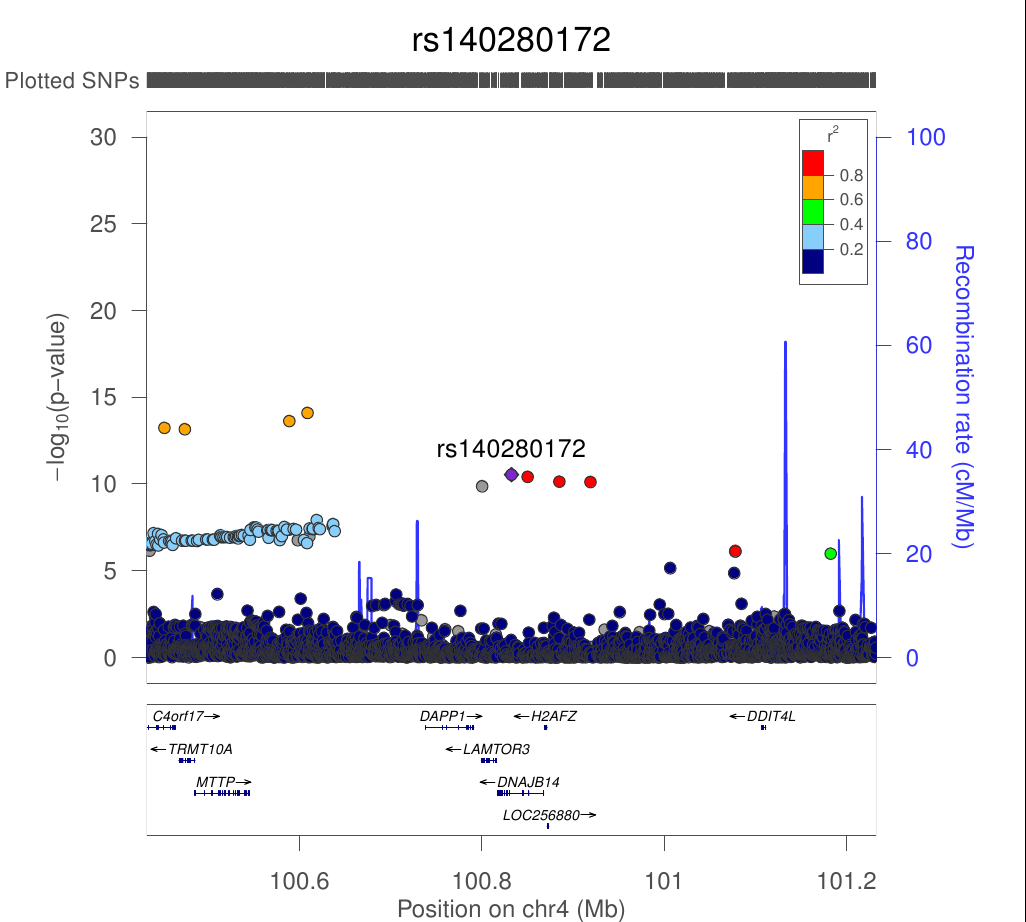


# Supplementary Figure 6: Locus Zoom plot of rs140280172 and LD with SNPs in the 400kb surrounding region.


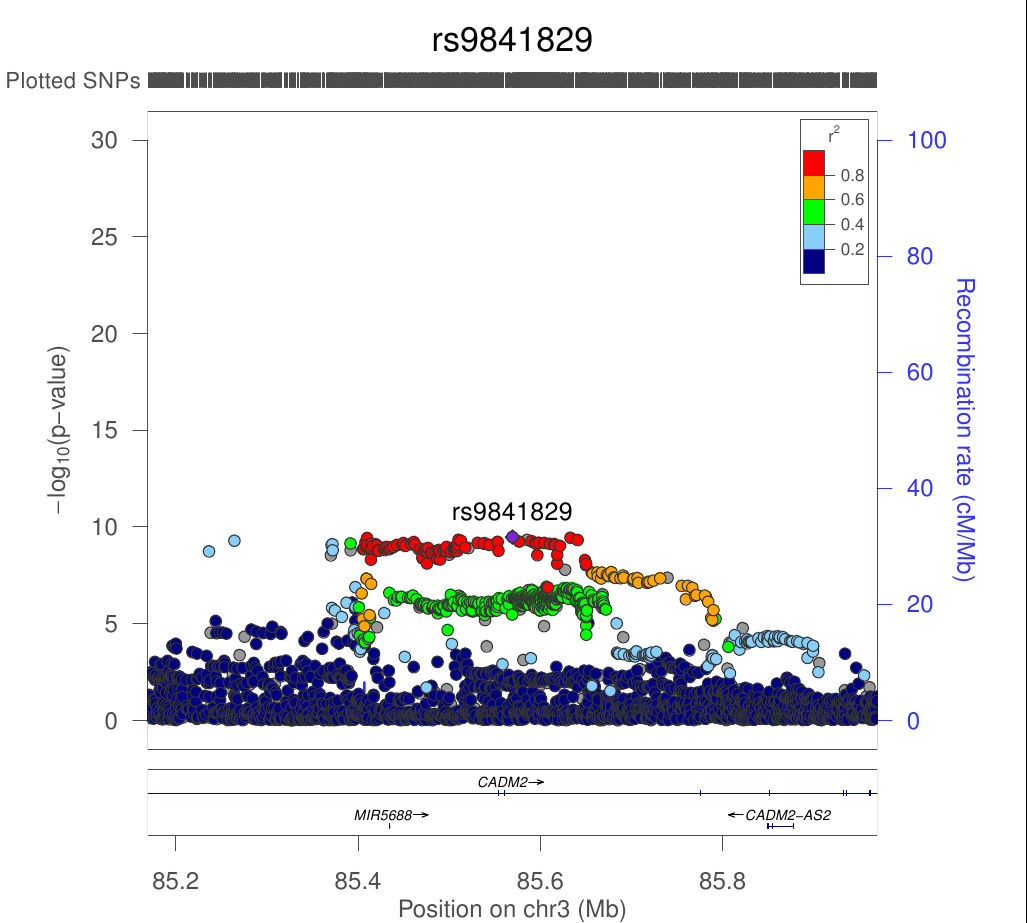


# Supplementary Figure 7: Locus Zoom plot of rs9841829 and LD with SNPs in the 400kb surrounding region.


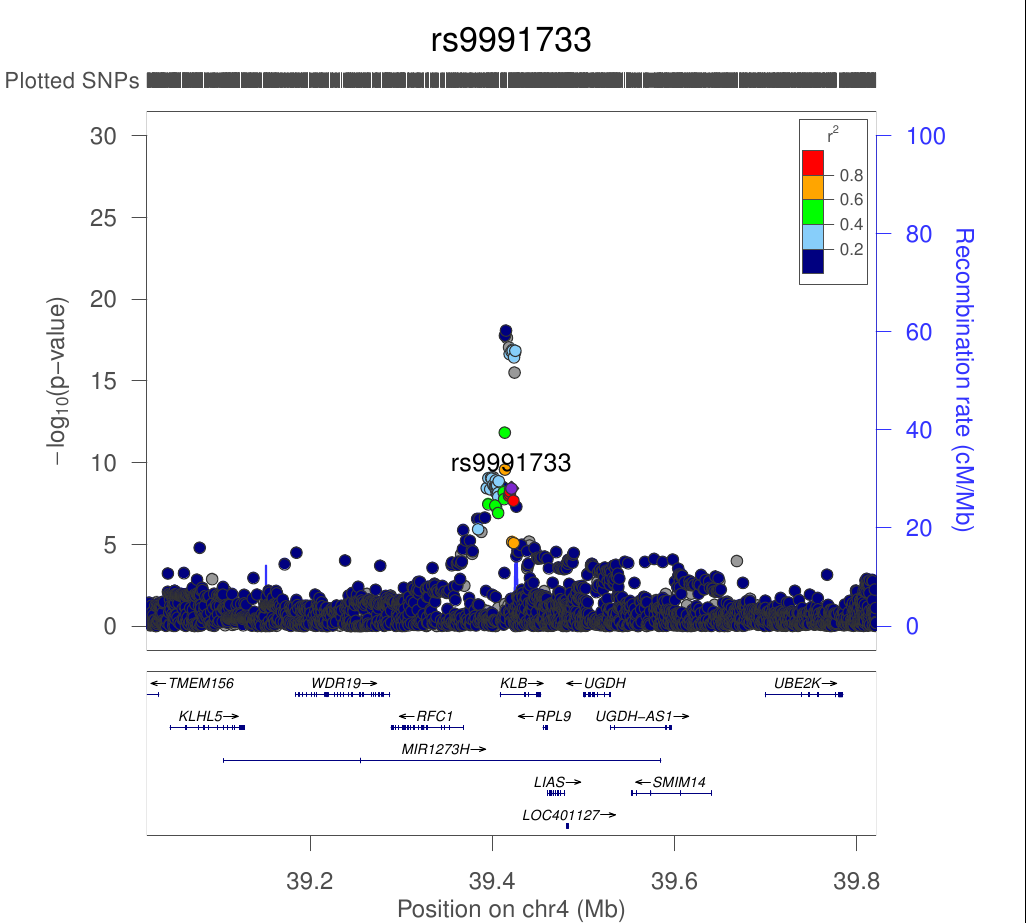


# Supplementary Figure 8: Locus Zoom plot of rs9991733 and LD with SNPs in the 400kb surrounding region.


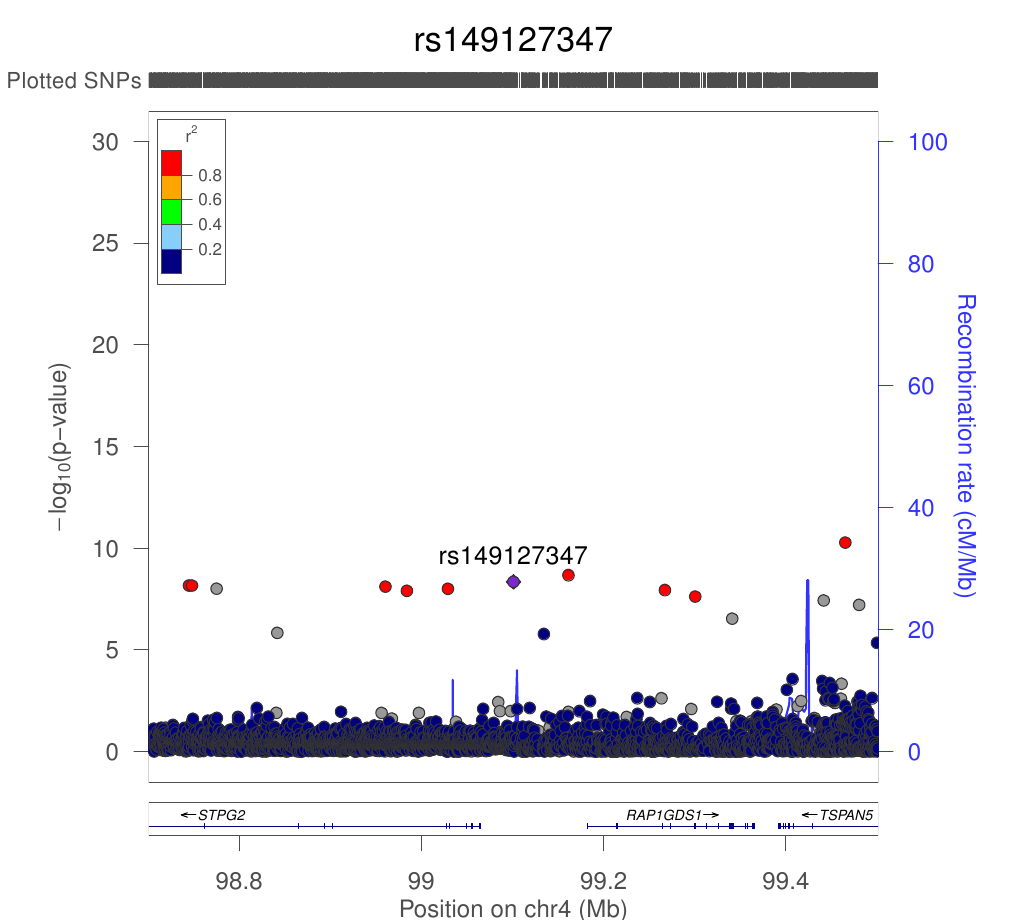


# Supplementary Figure 9: Locus Zoom plot of rs149127347 and LD with SNPs in the 400kb surrounding region.


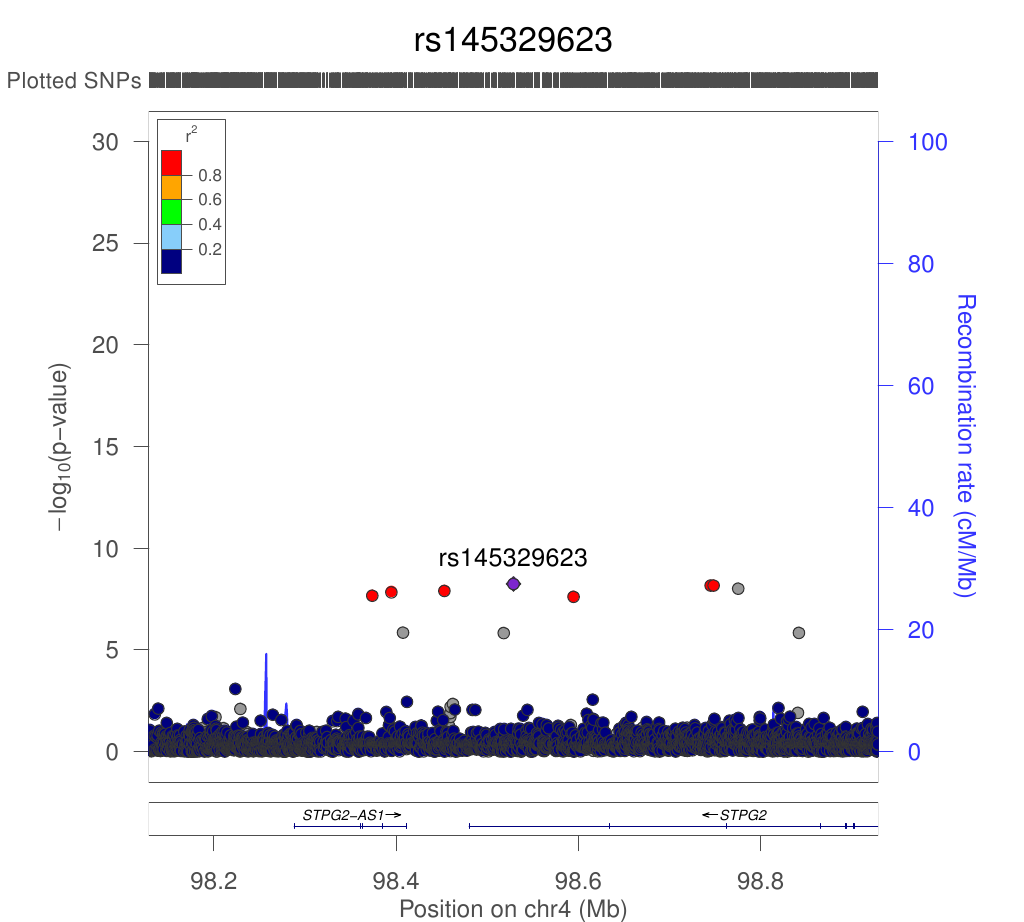


# Supplementary Figure 10: Locus Zoom plot of rs145329623 and LD with SNPs in the 400kb surrounding region.


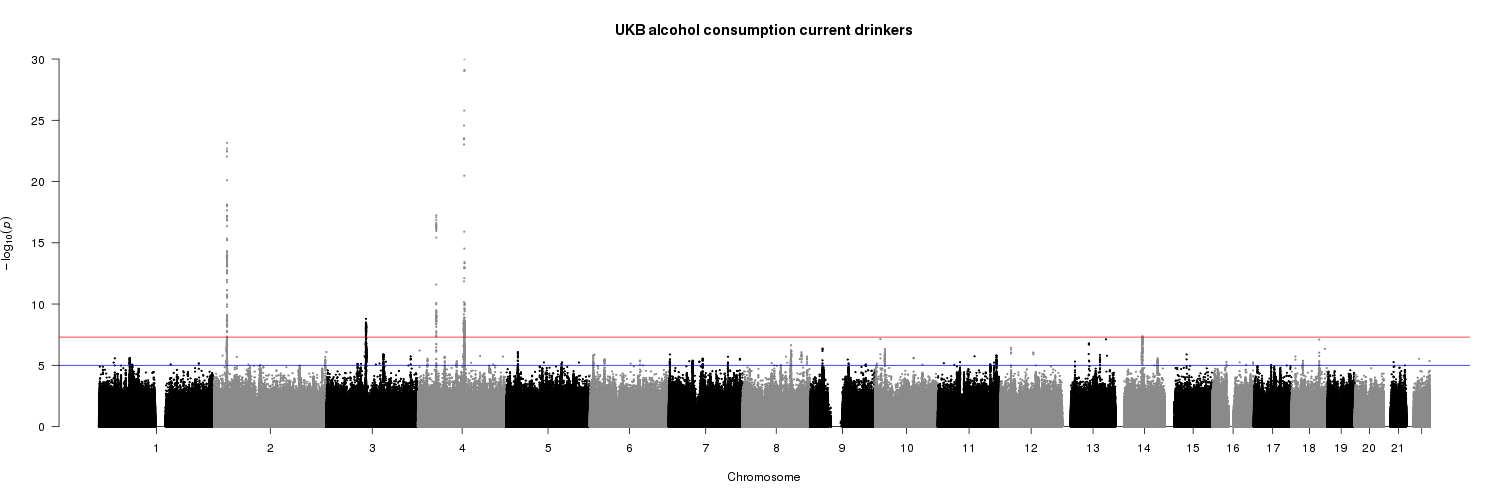


Supplementary Figure 11: Manhattan plot of alcohol consumption in current drinkers only in UKB (N=108,309). The red line shows the threshold for genome-wide significance (p ≤ 5 x 10-8) and the blue line shows the threshold for nominal significance (p ≤ 5 x 10-6)


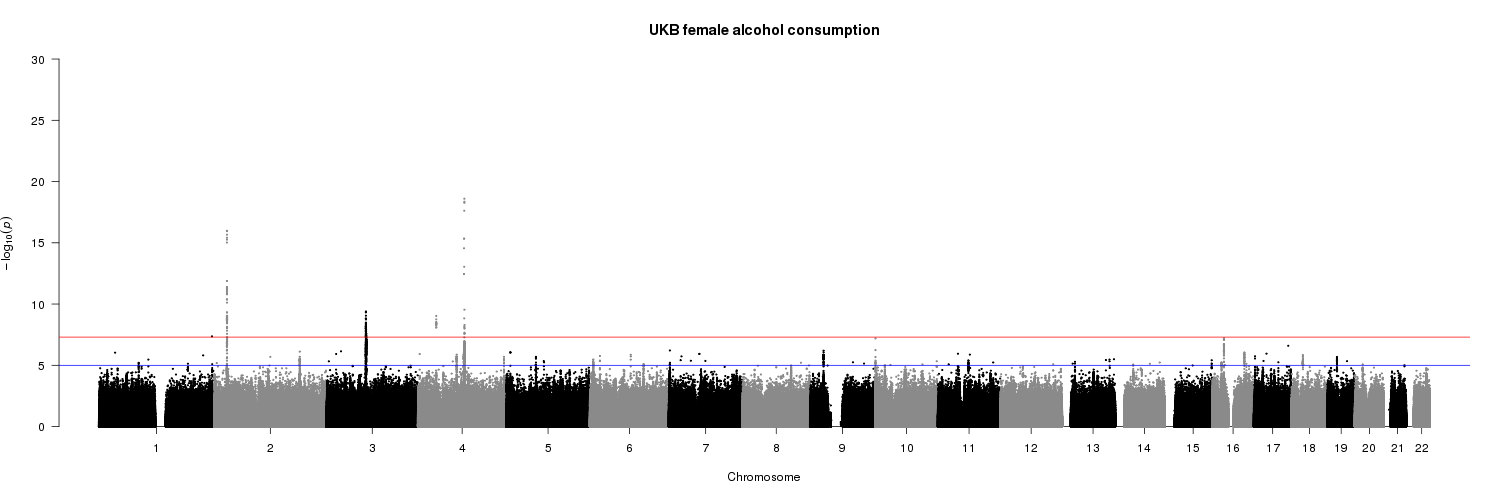


Supplementary Figure 12: Manhattan plot of alcohol consumption in females in UKB (N=59,088). The red line shows the threshold for genome-wide significance (p ≤ 5 x 10-8) and the blue line shows the threshold for suggestive significance (p ≤ 5 x 10-6)


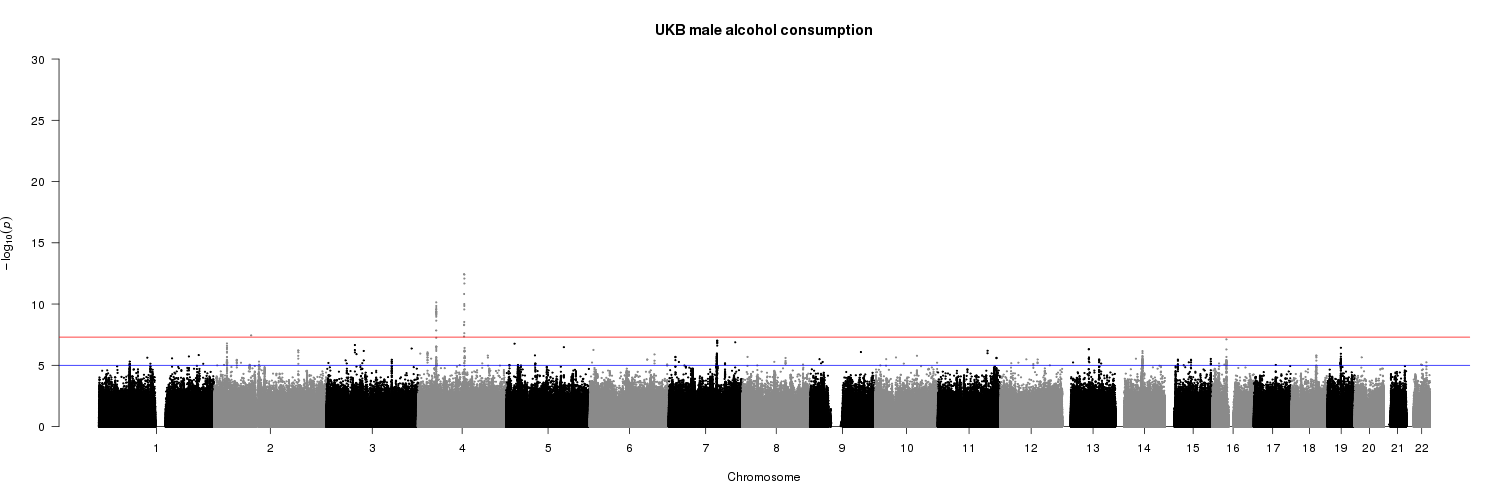


Supplementary Figure 13: Manhattan plot of alcohol consumption in males in UKB (N=53,089). The red lines show the threshold for genome-wide significance (p ≤ 5 x 10-8) and the blue line shows the threshold for suggestive significance (p ≤ 5 x 10-6).


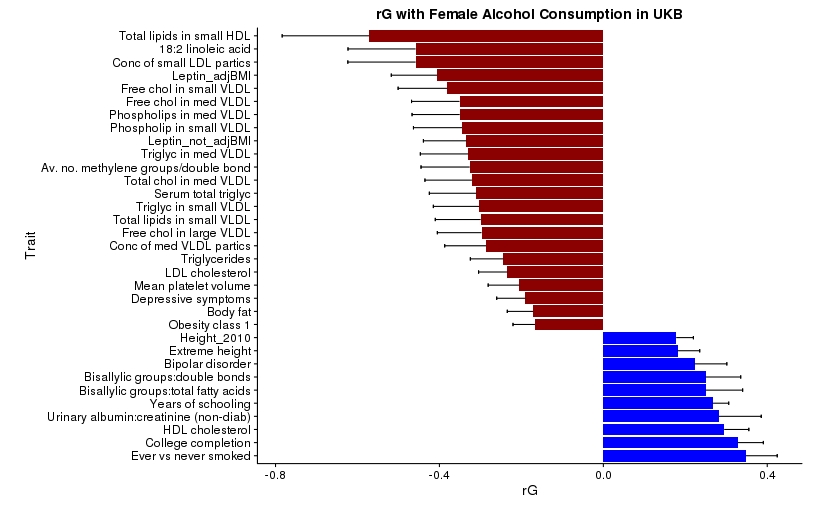


Supplementary Figure 14: Genetic correlation between female alcohol consumption in UKB and other traits using LD score regression implemented in LDHub. All traits presented were significant after correction for multiple testing, with the exception of bipolar disorder which was nominally significant after FDR correction (p≤0.06).


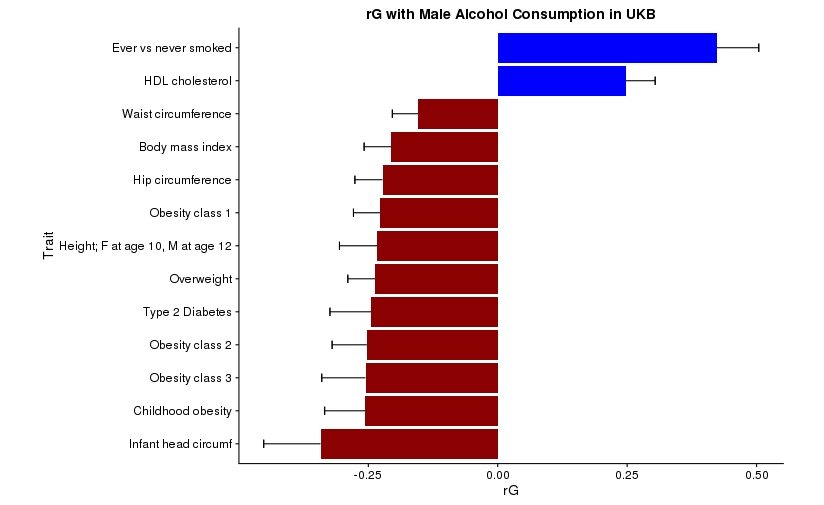


Supplementary Figure 15: Genetic correlation between male alcohol consumption in UKB and other traits using LD score regression implemented in LDHub. All traits presented were significant after correction for multiple testing.

| SNP | Original GWAS | adj  rs145329623 | adj  rs149127347 | adj  rs193099203 | adj  rs29001570 | adj  rs145452708 | adj  rs3114045 | adj  rs35081954 | adj  rs140280172 |
| --- | --- | --- | --- | --- | --- | --- | --- | --- | --- |
| rs145329623 | β=-0.017, p=5.68 x 10^-9^ |  | β=-0.017, p=0.29 | β=-0.005, p=0.14 | **β=-0.018, p=4.28 x 10^-9^** | β=-0.005, p=0.12 | β=-0.015, p=5.89 x 10^-7^ | **β=-0.017,**  **p=1.61 x 10^-8^** | β=-0.014, p=1.24 x 10^-6^ |
| rs149127347 | β=-0.018, p=4.42 x 10^-9^ | β=-0.0005, p=0.98 |  | β=-0.005, p=0.18 | **β=-0.018, p=3.33 x 10^-9^** | β=-0.005, p=0.12 | β=-0.015, p=4.81 x 10^-7^ | **β=-0.017, p=1.26 x 10^-8^** | β=-0.015, p=1.07 x 10^-6^ |
| rs193099203 | β=-0.031, p=3.79 x 10^-25^ | **β=-0.028, p=3.52 x 10^-17^** | **β=-0.028, p=5.22 x 10^-17^** |  | **β=-0.031, p=1.22 x 10^-25^** | β=-0.012, p=0.006 | **β=-0.028, p=4.24 x 10^-20^** | **β=-0.031, p=2.86 x 10^-24^** | **β=-0.028, p=7.45 x 10^-17^** |
| rs29001570 | β=-0.026, p=9.58 X 10^-19^ | **β=-0.027, p=2.50x 10^-19^** | **β=-0.027, p=2.94 x 10^-19^** | **β=-0.027, p=6.63 x 10^-19^** |  | **β=-0.026, p=1.43 x 10^-18^** | **β=-0.024, p=6.11 x 10^-15^** | **β=-0.026, p=1.38 x 10^-18^** | **β=-0.026, p=1.13 x 10^-18^** |
| rs145452708 | β=-0.034, p=1.15 x 10^-30^ | **β=-0.032, p=3.10 x 10^-24^** | **β=-0.032, p=3.67 x 10^-24^** | **β=-0.026, p=3.53 x 10^-10^** | **β=-0.034, p=1.28 x 10^-30^** |  | **β=-0.031, p=5.49 x 10^-24^** | **β=-0.033, p=5.01 x 10^-29^** | **β=-0.034, p=1.14 x 10^-21^** |
| rs3114045 | β=-0.020, p=7.98 x 10^-12^ | **β=-0.019, p=1.87 x 10^-10^** | **β=-0.019, p=1.70 x 10^-10^** | β=-0.015, p=5.72 x 10^-7^ | β=-0.016, p=3.02 x 10^-7^ | β=-0.012, p=7.36 x 10^-5^ |  | β=-0.016, p=2.05 x 10^-7^ | **β=-0.018, p=2.26 x 10^-9^** |
| rs35081954 | β=0.018, p=2.14 x 10^-9^ | **β=0.017, p=7.87 x 10^-9^** | **β=0.017, p=1.12 x 10^-8^** | β=0.016, p=1.32 x 10^-7^ | β=0.016, p=6.18 x 10^-8^ | β=0.015, p=4.75 x 10^-7^ | β=0.013, p=7.70 x 10^-5^ |  | **β=0.017, p=9.51 x 10^-9^** |
| rs140280172 | β=-0.020, p=2.89 X 10^-11^ | **β=-0.018, p=6.01 x 10^-9^** | **β=-0.017, p=7.71 x 10^-9^** | β=-0.007, p=0.03 | **β=-0.020, p=2.74 x 10^-11^** | β=-0.0003, p=0.94 | **β=-0.017, p=1.39 x 10^-8^** | **β=-0.020, p=2.19 x 10^-10^** |  |

Supplementary Table 1: Conditional GWAS analyses of SNPs associated with alcohol consumption on chromosome 4q. Bold highlighted p-values are genome-wide significant after adjusting for additional SNP covariates.

| **SNP** | **CHR** | **POS** | **A1/A2** | **Freq** | **β(se)** | **P** | **Genes** |
| --- | --- | --- | --- | --- | --- | --- | --- |
| rs145452708 | 4 | 100248642 | C/G | 0.01 | -0.035 (0.003) | 9.94 x 10^-31^ | *ADH1B/ADH1C* |
| rs193099203 | 4 | 99630017 | T/C | 0.007 | -0.032 (0.003) | 2.63 x 10^-25^ | *-* |
| rs11940694 | 4 | 39414993 | A/G | 0.39 | -0.027 (0.003) | 5.68 x 10^-18^ | *KLB* |
| rs29001570 | 4 | 99994405 | C/T | 0.006 | -0.025 (0.003) | 1.24 x 10^-16^ | *ADH5* |
| rs140280172 | 4 | 100832564 | A/C | 0.005 | -0.020 (0.003) | 8.49 x 10^-11^ | *DNAJB14* |
| rs3114045 | 4 | 100252560 | T/C | 0.13 | -0.019 (0.003) | 2.48 x 10^-10^ | *ADH1B/ADH1C* |
| rs9991733 | 4 | 39420994 | G/A | 0.28 | 0.019 (0.003) | 8.42 x 10^-10^ | *KLB* |
| rs2298755 | 4 | 100261038 | G/C | 0.41 | 0.019 (0.003) | 1.26x 10^-9^ | *ADH1C* |
| rs149127347 | 4 | 99101007 | G/T | 0.003 | -0.018 (0.003) | 2.16 x 10^-9^ | *-* |
| rs145329623 | 4 | 98528709 | G/A | 0.003 | -0.018 (0.003) | 3.84 x 10^-9^ | *STPG2* |
| rs1260326 | 2 | 27730940 | G/A | 0.38 | -0.03 (0.003) | 6.84 x 10^-24^ | *GCKR* |
| rs13078384 | 3 | rs13078384 | A/G | 0.31 | 0.018 (0.003) | 1.62 x 10^-9^ | *CADM2* |
| rs8012947 | 14 | 58784455 | A/G | 0.28 | 0.017 (0.003) | 4.25 x 10^-8^ | *ARID4A* |

Supplementary Table 2: Thirteen loci reaching genome-wide significance for association with alcohol consumption in current drinkers (N=108,309) in UKB. Genes are reported if located +/- 10kb of the locus.

| **Males** | | | | | | | |
| --- | --- | --- | --- | --- | --- | --- | --- |
| **SNP** | **CHR** | **POS** | **A1/A2** | **Freq** | **β(se)** | **P** | **Genes** |
| rs114026228 | 4 | 99567918 | C/T | 0.005 | -0.032 (0.004) | 3.60 x 10^-13^ | *TSPAN5* |
| rs145452708 | 4 | 100248642 | C/G | 0.01 | -0.031 (0.004) | 3.93 x 10^-13^ | *ADH1B/ADH1C* |
| rs28712821 | 4 | 39413780 | A/G | 0.40 | -0.029 (0.004) | 7.24 x 10^-11^ | *KLB* |
| rs29001570 | 4 | 99994405 | C/T | 0.006 | -0.028 (0.004) | 1.0 x 10^-10^ | *ADH5* |
| rs140089781 | 2 | 80079711 | A/G | 0.001 | -0.024 (0.004) | 3.58 x 10^-8^ | *CTNNA2* |
| **Females** | | | | | | | |
| rs145452708 | 4 | 100248642 | C/G | 0.01 | -0.037 (0.004) | 2.52 x 10^-19^ | *ADH1B/ADH1C* |
| rs144198753 | 4 | 99713350 | T/C | 0.008 | -0.033 (0.004) | 4.64 x 10^-16^ | - |
| rs11127048 | 2 | 27752463 | G/A | 0.38 | -0.035 (0.004) | 1.05 x 10^-16^ | *GCKR* |
| rs11940694 | 4 | 39414993 | A/G | 0.39 | -0.026 (0.004) | 9.31 x 10^-10^ | *KLB* |
| rs29001570 | 4 | 99994405 | C/T | 0.006 | -0.025 (0.004) | 1.48 x 10^-9^ | *ADH5* |
| rs3114045 | 4 | 100252560 | T/C | 0.13 | -0.024 (0.004) | 8.35 x 10^-9^ | *ADH1B/ADH1C* |
| rs1376935 | 3 | 85236425 | A/G | 0.32 | 0.026 (0.004) | 4.01 x 10^-10^ | *CADM2* |
| rs67028245 | 3 | 85394772 | A/G | 0.40 | -0.023 (0.004) | 3.74 x 10^-8^ | *CADM2* |

Supplementary Table 3: Loci reaching genome-wide significance for association with alcohol consumption in UKB in males and females only. Genes are reported if located +/- 10kb of the locus.

| **CHR** | **START** | **STOP** | **NSNPS** | **NPARAM** | **N** | **ZSTAT** | **P** | **GENE** |
| --- | --- | --- | --- | --- | --- | --- | --- | --- |
| 3 | 85008133 | 86123579 | 3965 | 93 | 111217 | 7.7284 | 5.44E-15 | *CADM2* |
| 4 | 39408473 | 39453153 | 182 | 44 | 110340 | 7.3013 | 1.43E-13 | *KLB* |
| 2 | 27719470 | 27746556 | 79 | 19 | 111080 | 7.1052 | 6.01E-13 | *GCKR* |
| 2 | 27805836 | 27846082 | 109 | 20 | 111051 | 6.7308 | 8.44E-12 | *ZNF512* |
| 2 | 27593363 | 27600400 | 20 | 9 | 111234 | 6.5109 | 3.73E-11 | *SNX17* |
| 2 | 27548716 | 27580243 | 43 | 16 | 110926 | 6.3232 | 1.28E-10 | *GTF3C2* |
| 4 | 1E+08 | 1E+08 | 124 | 9 | 111197 | 6.1647 | 3.53E-10 | *ADH1C* |
| 14 | 58765103 | 58840713 | 178 | 38 | 111192 | 6.045 | 7.47E-10 | *ARID4A* |
| 2 | 27667240 | 27712575 | 126 | 24 | 111653 | 6.0361 | 7.89E-10 | *IFT172* |
| 11 | 1.13E+08 | 1.13E+08 | 225 | 41 | 111218 | 5.946 | 1.37E-09 | *DRD2* |
| 14 | 58711523 | 58738727 | 70 | 19 | 110945 | 5.889 | 1.94E-09 | *PSMA3* |
| 2 | 27604066 | 27632550 | 76 | 17 | 111210 | 5.669 | 7.18E-09 | *PPM1G* |
| 2 | 27799389 | 27805589 | 16 | 6 | 110254 | 5.5328 | 1.58E-08 | *C2orf16* |
| 1 | 66258193 | 66840262 | 2016 | 133 | 110793 | 5.5251 | 1.65E-08 | *PDE4B* |
| 2 | 27477440 | 27501093 | 49 | 18 | 111256 | 5.4983 | 1.92E-08 | *SLC30A3* |
| 2 | 27714750 | 27718126 | 12 | 6 | 111593 | 5.3533 | 4.32E-08 | *FNDC4* |
| 2 | 27505297 | 27530307 | 48 | 15 | 111199 | 5.3451 | 4.52E-08 | *TRIM54* |
| 4 | 1E+08 | 1E+08 | 178 | 36 | 111610 | 5.3112 | 5.44E-08 | *C4orf17* |
| 2 | 27587219 | 27593324 | 17 | 11 | 111570 | 5.2684 | 6.88E-08 | *EIF2B4* |
| 7 | 1.41E+08 | 1.41E+08 | 1395 | 137 | 110788 | 5.1259 | 1.48E-07 | *TMEM178B* |
| 2 | 27851515 | 27873713 | 71 | 17 | 111363 | 5.1202 | 1.53E-07 | *GPN1* |
| 14 | 58666833 | 58702354 | 99 | 23 | 110359 | 5.1177 | 1.55E-07 | *ACTR10* |
| 6 | 4706393 | 4955778 | 955 | 126 | 110762 | 5.0826 | 1.86E-07 | *CDYL* |
| 7 | 51083909 | 51384515 | 963 | 83 | 111368 | 4.9763 | 3.24E-07 | *COBL* |
| 8 | 1.45E+08 | 1.45E+08 | 116 | 37 | 111084 | 4.9168 | 4.40E-07 | *SCRIB* |
| 14 | 57267425 | 57277194 | 14 | 7 | 108435 | 4.9023 | 4.74E-07 | *OTX2* |
| 17 | 44316744 | 44415160 | 107 | 21 | 108941 | 4.885 | 5.17E-07 | *LRRC37A* |
| 18 | 21086148 | 21166581 | 210 | 37 | 111302 | 4.8669 | 5.67E-07 | *NPC1* |
| 9 | 26840683 | 26892826 | 195 | 45 | 111167 | 4.8402 | 6.49E-07 | *CAAP1* |
| 2 | 27651473 | 27665126 | 29 | 8 | 111168 | 4.7798 | 8.77E-07 | *NRBP1* |
| 2 | 76974849 | 77749502 | 3528 | 138 | 110870 | 4.7746 | 9.00E-07 | *LRRTM4* |
| 17 | 44351550 | 44439416 | 50 | 22 | 108367 | 4.7723 | 9.11E-07 | *ARL17B* |
| 6 | 7281283 | 7313541 | 216 | 27 | 110977 | 4.7653 | 9.43E-07 | *SSR1* |
| 5 | 77781038 | 77944648 | 576 | 44 | 110911 | 4.7592 | 9.72E-07 | *LHFPL2* |
| 18 | 21083434 | 21113311 | 80 | 20 | 111321 | 4.705 | 1.27E-06 | *C18orf8* |
| 2 | 99771418 | 99779620 | 39 | 11 | 111852 | 4.6905 | 1.36E-06 | *LIPT1* |
| 16 | 72097125 | 72111145 | 59 | 18 | 111429 | 4.6101 | 2.01E-06 | *HPR* |
| 17 | 47074774 | 47133507 | 158 | 32 | 110350 | 4.564 | 2.51E-06 | *IGF2BP1* |
| 17 | 44107282 | 44302740 | 352 | 44 | 110926 | 4.5515 | 2.66E-06 | *KANSL1* |
| 2 | 27886338 | 27917847 | 59 | 18 | 111679 | 4.5505 | 2.68E-06 | *SLC4A1AP* |
| 11 | 47440320 | 47448024 | 25 | 9 | 110566 | 4.542 | 2.79E-06 | *PSMC3* |

Supplementary Table 4: Results of MAGMA gene-based association analyses. Only genes significant after correction for multiple testing presented (p <2.8 x 10-6)

| SNP | Location of SNP | Tissue | eQTL gene | GTEx  P-Value |
| --- | --- | --- | --- | --- |
| rs11940694 | *KLB* | Muscle skeletal | *RFC1* | 0.0000037 |
|  |  | Muscle skeletal | *RPL9* | 0.0000038 |
|  |  | Brain – Cerebellar hemisphere | *RFC1* | 0.000022 |
|  |  | Brain Cerebellum | *RFC1* | 0.000053 |
| rs1260326 | *GCKR* | Muscle Skeletal | *SNX17* | 1.7e-11 |
|  |  | Whole Blood | *NRBP1* | 3.9e-9 |
|  |  | Thyroid | *FNDC4* | 3.7e-8 |
|  |  | Adrenal Gland | KRTCAP3 | 0.0000032 |
|  |  | Testis | *NRBP1* | 0.0000084 |
|  |  | Adipose - Subcutaneous | *NRBP1* | 0.000011 |
|  |  | Skin (Subrapubic) | *NRBP1* | 0.000052 |
|  |  | Colon - Transverse | *NRBP1* | 0.000056 |
|  |  | Cells – Transformed Fibroblasts | *ATRAID* | 0.000061 |
|  |  | Thyroid | *GCKR* | 0.000088 |
|  |  | Tibial Nerve | *ATRAID* | 0.00016 |
| rs9991733 | *KLB* | Whole Blood | *UGDH* | 0.000011 |
| rs9841829 | *CADM2* | Lung | *CADM2* | 5.2e-9 |
|  |  | Adipose - subcutaneous | *CADM2* | 0.000023 |
|  |  | Adipose - visceral | *CADM2* | 0.000045 |

Supplementary Table 5: GTEx analysis of SNPs associated with alcohol consumption at genome-wide significance and shown to be an eQTL according to GTEx database.
